# Short-Term Tensiomyography Responses of the Vastus Medialis to Percussive Massage Therapy with Different Frequency–Duration Combinations

**DOI:** 10.3390/jfmk11020163

**Published:** 2026-04-21

**Authors:** Sara Ascic, Mijo Curic, Iva Sklempe Kokic

**Affiliations:** 1 Faculty of Economics Osijek, Josip Juraj Strossmayer University of Osijek, 31000 Osijek, Croatia; 2 Faculty of Kinesiology Zagreb, University of Zagreb, 10000 Zagreb, Croatia; 3 Faculty of Kinesiology Osijek, Josip Juraj Strossmayer University of Osijek, 31000 Osijek, Croatia

**Keywords:** sports performance recovery, neuromuscular fatigue, skeletal muscle contractile properties

## Abstract

**Background**: Percussive massage therapy (PMT) with handheld massage guns is widely used to support recovery and flexibility, but the short-term behavior of skeletal muscle contractile properties and the relative contribution of application duration versus frequency remain unclear. This study investigated the 10 min post-intervention time course of tensiomyography (TMG)-derived contractile properties of non-fatigued vastus medialis (VM) after clinically realistic PMT protocols and examined whether longer duration is associated with persistent deviations from baseline than frequency. **Methods**: In a two-session, within-subject repeated-measure design, 32 participants completed four PMT conditions to the VM (35 Hz–3 min, 35 Hz–6 min, 45 Hz–3 min, and 45 Hz–6 min). TMG parameters (Td, Tc, Ts, Tr, and Dm) were recorded at baseline and repeatedly over 10 min post-intervention. Linear mixed-effect models with frequency and duration as fixed factors and time as continuous and categorical were used to characterize temporal patterns, with emphasis on effect sizes and consistency across parameters. The fixed protocol order (35 Hz in session one, 45 Hz in session two, 3 vs. 6 min assigned to contralateral legs) means that frequency was confounded with session and duration with leg side. **Results**: Compared with the 3 min protocols, the 6 min protocols were associated with slightly higher Td and Ts, a modest increase in Tr and a slightly greater Dm (e.g., Dm + 0.55 mm), whereas Tc showed no clear duration effect. Across conditions, Td increased immediately after PMT, Tc remained elevated for most of the first 8 min, Ts increased from mid to late post-intervention, Tr changed inconsistently, and Dm was reduced relative to baseline for most of the 10 min period. Differences between 35 and 45 Hz were small and non-significant for all TMG parameters. **Conclusions**: Clinically realistic PMT protocols at 35–45 Hz in non-fatigued muscle induce small but statistically detectable, duration-sensitive changes in TMG-derived contractile behavior over approximately 10 min. Within the constraints of the fixed, non-randomized design and the small effect sizes observed, these findings support viewing massage gun use as a recovery-oriented adjunct that subtly modulates contractile dynamics, rather than as a strong, standalone performance-enhancing stimulus.

## 1. Introduction

Massage therapy is commonly described as the manual manipulation of superficial soft tissues, including skin, muscle, tendon, ligament and fascia for therapeutic purposes [1,2]. It is widely used to modulate pain in both clinical and sports populations, with systematic reviews generally reporting modest reductions in pain intensity and improvements in pain-related function, although the certainty of evidence is often low to moderate [1,3,4]. Beyond pain, massage is frequently applied to enhance post-exercise recovery and attenuate delayed onset muscle soreness [2,5,6] and has been identified as one of the more effective recovery modalities for reducing fatigue-related symptoms after exercise [7]. Massage interventions can also improve range of motion, particularly in the shoulder and lower limb, although the magnitude, time course and persistence of these effects vary across studies [2,5,8]. In addition, massage may contribute to improved mood [4] and small improvements in health-related quality of life in some populations [1].

Despite the long history of use and the broad range of reported benefits, findings regarding the effects of massage on performance-related and psychosocial outcomes remain heterogeneous, which may in part reflect the diversity of massage modalities, dosages and protocols used in practice [1,2,7]. Massage therapy encompasses a wide spectrum of techniques, most of which are delivered manually by a therapist, whereas in recent years device-based approaches, including vibration and percussion-type interventions, have emerged as distinct subgroups within the broader massage paradigm [2]. These newer methods are frequently marked and used as recovery tools after fatiguing exercise, yet the underlying neuromuscular mechanisms and dose–response relationships remain incompletely understood.

Among device-based massage approaches, percussive massage therapy (PMT) delivered by handheld massage guns has become increasingly common in sport and exercise settings, particularly as an accessible form of self-myofascial treatment [9,10,11,12]. These devices enable users to self-administer treatment and adjust parameters such as frequency, pressure and treatment area according to individual tolerance and needs, which likely contributes to their rapid uptake in both training and rehabilitation environments [9,13]. PMT is proposed to act on deeper soft tissues with goals such as reducing pain, decreasing muscle tone and stiffness, increasing joint mobility and facilitating recovery, although many of the underlying mechanisms, such as changes in fascial gliding, neuromodulation of pain, or alterations in muscle spindle and Golgi tendon organ activity, remain theoretical and partially supported by empirical data [11,14]. Recent work comparing PMT with other recovery modalities, such as foam rolling, further suggests that percussion can influence markers of delayed onset muscle soreness and perceived recovery, but responses remain variable across protocols, muscles and populations [11,12].

Acute studies show that PMT can increase flexibility and joint range of motion and reduce passive stiffness in the lower limb, typically without concomitant improvements in maximal strength [9,15]. Its effects on hamstring extensibility appear similar in magnitude to those of static stretching or muscle energy techniques [16,17]. In athletes and patients with musculoskeletal conditions, PMT used as an adjunct to conventional care has been associated with improvements in pain, spinal or joint mobility, proprioception and functional outcomes, although effect sizes and responder rates vary considerably [18,19,20]. Multi-session protocols with progressively increasing frequency can yield meaningful gains in hip flexion and ankle dorsiflexion range of motion, suggesting that PMT can be programmed to target flexibility adaptations over time [12]. By contrast, evidence for benefits on neuromuscular performance and recovery is inconsistent. Several studies report no acute enhancement when PMT is applied during warm-up or immediately after fatiguing exercise [21,22,23,24,25], whereas using PMT during inter-set rest in resistance training has been shown to attenuate movement velocity loss in bench press [13]. Taken together, current evidence indicates that massage guns can meaningfully modify flexibility, stiffness, pain and perceived recovery, but their effects on strength and explosive performance appear highly dependent on treatment parameters, timing relative to exercise and population studied [11,12]. Because many of these observed changes may reflect alterations in underlying muscle contractile behavior, objective in vivo measures of contractile properties may help clarify the mechanisms and time course of PMT effects in both non-fatigued and fatigued muscle.

In the context of exercise and recovery, it is useful to distinguish between non-fatigued and fatigued muscle states. In non-fatigued muscle, tensiomyography (TMG)-derived parameters primarily reflect intrinsic contractile characteristics such as stiffness, contraction speed, sustainment of contraction and relaxation dynamics. In fatigued muscle, these parameters are additionally influenced by reduced force-generating capacity, altered excitation–contraction coupling and metabolite accumulation, which modify the time course and magnitude of contractile responses [26,27].

Tensiomyography (TMG) is a non-invasive method for assessing the contractile properties of superficial skeletal muscles in vivo, by quantifying radial muscle belly displacement and time-related parameters in response to an electrically evoked twitch [28]. TMG shows high test–retest and inter-rater reliability for key parameters such as maximal displacement (Dm), contraction time (Tc) and delay time (Td), whereas reliability indices for sustain time (Ts) and relaxation time (Tr) are generally lower and more variable across studies, suggesting Ts and Tr should be interpreted with great caution [27,29]. Recent work also indicates good diagnostic accuracy of TMG-derived parameters for monitoring peripheral neuromuscular fatigue, supporting its practical use in both research and applied sport settings [26]. Given that massage guns aim to modify muscle tone, stiffness and fatigue-related properties through high-frequency mechanical stimulation, a method that is sensitive to small changes in contractile behavior is well-suited to quantify their acute effects [9,11]. However, while TMG has been widely used to characterize fatigue and recovery kinetics after exercise, the short-term course of TMG responses to PMT itself and the extent to which different frequency–duration combinations differentially shape this time course has not been systematically described.

Recently, we conducted a crossover study in which participants received PMT of the vastus medialis (VM) at different frequencies and duration and muscle contractile properties were assessed with TMG immediately before and after interventions [30]. That analysis showed that higher frequencies and longer duration produced significant changes in several TMG-derived variables, indicating that both parameters influence the acute TMG response to massage gun application when assessed at a single post-intervention time point. However, measurements were limited to one post-intervention assessment, so it remained unclear whether these effects normalized rapidly, changed further during the immediate recovery period or remained relatively stable over a short post-treatment window [30]. In the context of muscle fatigue and recovery, understanding whether PMT-induced contractile changes dissipate within minutes or persist over a relevant time frame is important for practical decisions about the timing of PMT relative to fatiguing tasks, warm-up routines and competitions.

Therefore, the aim of this study was to explore the short-term time course of changes in TMG-derived contractile properties following different PMT conditions by analyzing all available post-intervention measurements over a 10 min period in non-fatigued muscle. We expected that different combinations of PMT frequency and duration would be associated with small but systematic deviations from baseline contractile behavior over the observation window, rather than identical responses across all protocols. Since in the present design frequency was linked to session order and duration to leg side and leg order, any contrasts between these factors must be interpreted with caution and in the context of potential session or side-related influences. Within these design constraints, we anticipated that longer application durations would be more likely than shorter ones to coincide with more persistent deviations from baseline, whereas differences between 35 and 45 Hz were expected to be modest. Given the exploratory nature of several secondary outcomes and the limited prior data on TMG time courses after PMT, these expectations were specified in terms of relative trends in the magnitude and persistence of changes rather than a priori time-specific contrasts. By providing a detailed description of the early post-intervention behavior of TMG parameters in response to clinically realistic PMT protocols, we sought to offer mechanistic, contractile-level insights that can inform future work linking massage gun use to neuromuscular fatigue, recovery and functional performance, while explicitly recognizing that functional implications cannot be directly inferred from TMG alone.

## 2. Materials and Methods

### 2.1. Design

The study used a two session, within-subject repeated-measure design in which all participants completed four PMT conditions (35 Hz–3 min, 35 Hz–6 min, 45 Hz–3 min and 45 Hz–6 min). Both testing sessions were conducted at the Faculty of Kinesiology, University of Osijek, under standardized environmental conditions between 09:00 and 14:00 h and were scheduled at least 5 days apart to reduce potential carry-over effects. Time point 0 denoted baseline, time point 1 denoted the first post-intervention measurement immediately after PMT, and time points 2–21 successive post-intervention measurements at 30 s intervals up to 10 min, yielding 22 time points per condition. All TMG parameters (Td, Tc, Ts, Tr and Dm) were recorded at each time point, with Tc and Dm considered the primary outcomes a priori based on their widespread use and superior reliability for monitoring neuromuscular fatigue and stiffness and Td, Ts and Tr treated as secondary parameters that provide complementary but more error-prone information. Randomization of condition order and blinding of the TMG assessor were not implemented; instead, all participants received 35 Hz protocols in the first session and 45 Hz protocols in the second session and within each session the right leg was always assessed before the left, with the 3 and 6 min durations assigned in a fixed manner to contralateral legs. This fixed structure was chosen to maintain tightly controlled measurement conditions and to minimize procedural variability for repeated TMG assessments; however, it also introduced systematic confounding of frequency with session order and of duration with leg side and leg order. Accordingly, the present comparison between frequency and duration should be interpreted as exploratory and design-dependent rather than as a clean causal contrast. Because TMG parameters are objective, instrument-derived measures, the risk of observed bias is likely attenuated but not eliminated, and potential effects of fatigue, learning or subtle changes in positioning or tissue state between sessions cannot be entirely ruled out. The absence of randomization, assessor blinding and a non-PMT control or sham condition should therefore be borne in mind when interpreting the frequency and duration effects and when generalizing the present frequency–duration patterns. Participants were instructed to avoid vigorous physical activity during the 72 h before each session and reported no events between sessions that could influence muscle function. No additional warm-up was performed to isolate the acute effects of the massage gun interventions on (VM) contractile properties.

### 2.2. Participants

Participants were 32 healthy students (15 female, 17 male) from the Faculty of Kinesiology, University of Osijek, recruited as a convenience sample. Their mean ± standard deviation age, height and body mass were 22.81 ± 1.93 years, 177.30 ± 9.29 cm and 72.17 ± 13.41 kg, respectively. All students were regularly engaged in sport and physical activity as part of their study program and had previous training experience in at least one sport. Inclusion criteria were current enrolment as a kinesiology student and regular participation in organized or structured physical activity. Exclusion criteria were any lower-limb injury or musculoskeletal complaint in the previous 6 months or other health problems that could influence neuromuscular function. Participants were recruited via in-class invitations during lectures and word of mouth. Thirty-six students attended the initial session and four were excluded because they reported vigorous physical activity between testing sessions, resulting in a final sample of 32 participants who completed all measurements. The study protocol was approved by the Ethics Committee of the Faculty of Kinesiology, University of Osijek (classification mark 029-01/24-01/05 and registration number 2158-110-01-24-73), and all procedures were conducted in accordance with the Declaration of Helsinki. All participants provided written informed consent prior to participation.

### 2.3. Tensiomyography

TMG was used to assess skeletal muscle contractile properties of the VM. Measurements were obtained from the vastus medialis of both legs using a TMG device (TMG-BMC, Ljubljana, Slovenia) and a digital displacement sensor (GK40, Panoptik, Ljubljana, Slovenia). Participants lay supine on a massage table with the knee supported in 30° flexion using the manufacturer’s wedge cushion and this position was replicated at every measurement. Electrode and sensor placement followed the manufacturer’s recommendations and previous studies assessing the VM with TMG [31,32,33]. The measurement point was located at 80% of the line between the anterior iliac spine and the anterior border of the medial collateral ligament, with the sensor positioned almost perpendicular to this line, and participants were instructed to extend the knee against manual resistance to confirm muscle activation. Single stimulus square-wave pulses (1 ms) were delivered via a constant-current stimulator, starting at 20 mA and increased in 10 mA increments until no further increase in maximal radial displacement was observed, plus an additional 10 mA to ensure supramaximal stimulation. At baseline (time point 0) and immediately post-intervention (time point 1), 3–5 twitches were recorded and the best curve was selected; for subsequent time points, a single supramaximal twitch was recorded at the same stimulation intensity. This approach follows common TMG practice in which multiple twitches are used at key time points to optimize signal quality, while single twitches are employed for dense repeated sampling to limit participant burden and measurement time. Nevertheless, using several twitches at baseline and immediately post-intervention may have reduced random variability at these time points compared with later measurements, and this potential influence of measurement strategy on early versus late pattern is considered when interpreting the time course. From the selected twitch responses, Td, Tc, Ts, Tr and Dm were extracted according to standard definitions. Piqueras-Sanchiz et al. [34] reported that tensiomyography of the VM shows excellent reliability for Dm and Tc and good to excellent reliability for Td, with intraclass correlation coefficient between 0.93 and 0.99, and low coefficient of variation between 1.8 and 3.8%. In contrast, Tr demonstrated moderate to excellent relative reliability (intraclass correlation coefficients ranging between 0.82 and 0.86), but substantially higher measurement error, with coefficient of variation ranging from 18.8 to 40.9%. Similarly, Ts showed good to excellent relative reliability (intraclass correlation coefficients ranging from 0.83 to 0.96), accompanied by higher measurement error, with coefficient of variation ranging from 12.7 to 22.9%. To provide a comprehensive characterization of muscle contractile properties, all standard TMG-derived parameters were analyzed, as they capture complementary aspects of muscle stiffness, contraction speed, contraction maintenance and relaxation dynamics, although results for Ts and Tr were interpreted with particular caution due to their higher measurement error. Because the present study focused on relatively small acute changes in these parameters, their known reliability characteristics were considered when interpreting the magnitude and potential practical relevance of the observed effects.

### 2.4. Percussion Therapy Intervention

Percussive stimulation was applied to the VM using a handheld massage gun (Hypervolt 2 pro, Hyperice Inc., Irvine, CA, USA) equipped with a round ball attachment, a model that is commonly used in clinical settings and has been used in several studies [9,16,23]. The device was applied directly to the skin over the muscle with moderate pressure, standardized using the built-in three light pressure indicator and maintained at the level corresponding to a single illuminated light. Two frequencies (35 and 45 Hz, approximately 2100 and 2700 percussion per minute, respectively) and two continuous application durations (3 and 6 min) were combined to form four percussion protocols. A licensed physiotherapist with experience in manual and percussive therapies delivered all interventions, using slow sweeping strokes along the muscle fibers from distal to proximal and back to distal, completing each distal–proximal–distal cycle in approximately 20 s and repeating continuously for the prescribed duration. This protocol was chosen to reflect clinically realistic recovery-oriented applications while allowing systematic manipulation of frequency and duration. Although pressure was standardized using the device’s three-light indicator and delivered by a single experienced physiotherapist, small between-trial and between-leg variations in applied pressure and stroke pattern are likely and may have contributed to within-subject variability.

### 2.5. Experimental Protocol

Each participant completed two sessions separated by at least 5 days. At the beginning of each session, TMG of the VM was performed at baseline (time point 0) on both legs, with the right leg always assessed before the left. In the first session, participants received the 35 Hz percussive protocol, with a 3 min intervention on one leg and a 6 min intervention on the contralateral leg. In the second session, they received the 45 Hz protocols in the same manner. Participants were instructed to avoid vigorous physical activity during the 72 h before each session and confirmed compliance on arrival. For each leg, the percussive intervention was immediately followed by repeated TMG measurements at time points 1–21 (time point 1 immediately post-intervention, while time points 2–21 were 30 s apart), before moving on to the contralateral leg within the same session. During the massage gun application, participants remained supine on the same table without the knee support cushion. After percussion intervention, the cushion was repositioned and the 30° knee angle rechecked before each subsequent TMG assessment to ensure consistent measurement conditions. Leg dominance was not explicitly modeled in the analysis, and the fixed order of leg assessments within each session may have introduced systemic influences (e.g., subtle fatigue, learning or positional changes) and should therefore be considered when interpreting side-to-side differences.

### 2.6. Statistical Analysis

An a priori power analysis using G*Power 3.1.9.4, based on a repeated-measure ANOVA with one within-subject factor (four levels), effect size f = 0.25, α = 0.05, power = 0.90, correlation among repeated measures = 0.50 and ε = 1.0, indicated that a minimum of 30 participants would be required to detect medium within-subject effects of condition. Because G*Power does not currently provide a dedicated procedure for a priori sample size estimation for linear mixed-effect models (LMMs) with many repeated time points, repeated-measure ANOVA was used as an approximation to the planned within-subject structure. As such, the resulting target focuses on effects of condition averaged across time, whereas smaller effects, higher-order interactions and exploratory secondary outcomes are likely to be relatively underpowered. The final sample of 32 participants was therefore considered sufficient to provide reasonably precise estimates of main within-subject effects, while acknowledging that smaller effects and complex interactions, particularly for secondary outcomes, may be underpowered. All subsequent statistical analyses were conducted in SPSS (version 20, IBM Corp., Armonk, NY, USA). Descriptive statistics were calculated for all TMG parameters at baseline for each percussion condition. Baseline differences between conditions were additionally evaluated using LMMs restricted to time 0, with frequency (35 vs. 45 Hz) and duration (3 vs. 6 min) entered as fixed factors and a random intercept for subject. These baseline models were used to determine whether TMG parameters differed between percussion conditions at time 0, so that subsequent time effects could be interpreted primarily as within-condition changes over time, and duration-related findings are viewed as patterns of change within this fixed design rather than as pure duration effects.

For the primary analyses, LMMs were fitted to the full data set, with frequency and duration specified as fixed experimental factors and time as a repeated-measure factor within each-subject condition. All models included a random intercept for subject and an autoregressive (AR(1)) covariance structure for the repeated measurements over time within each subject condition, reflecting the expectation that temporally adjacent measurements are more strongly correlated than distant ones. Time was first modeled as a continuous covariate to examine whether TMG parameters changed approximately linearly over the observation period and whether these linear trends differed by frequency and duration (time × frequency, time × duration, and frequency × duration × time interactions). This continuous-time specification was chosen to capture overall tendencies in the post-intervention course and to summarize frequency- and duration-related trends across the entire window. For interpretability, estimated marginal means (EMMs) for frequency and duration (averaged over time) were obtained and levels within each factor were compared using Bonferroni-adjusted pairwise tests, with particular emphasis on Tc and Dm as primary outcomes. Secondary models treated time as a categorical factor (levels 0–21) to relax the linearity assumption and to identify the specific post-intervention time points at which TMG parameters differed from baseline (Time 0), using Bonferroni-adjusted pairwise comparisons of EMMs between baseline and each subsequent time level. This categorical-time approach complements the continuous models by allowing inspection of non-linear and transient patterns that may not be fully captured by a single linear term. Given the exploratory nature of the multiple TMG outcomes and the large number of time points, emphasis was placed on the magnitude and consistency of effect sizes and temporal patterns across parameters rather than on the statistical significance of individual comparisons. For all LMMs, type III tests of fixed effects are reported together with partial eta-squared (η^2^p) as an effect size measure, calculated from the model sums of squares as SSeffect/(SSeffect + SSerror) and interpreted as small (0.01), moderate (0.06) or large (0.14) [35]. For pairwise comparisons, Cohen’s d is additionally reported, calculated as the mean difference divided by the pooled standard deviation of the two levels being compared. Statistical significance was set at *p* < 0.05 (two-tailed) but these *p* values are interpreted alongside effect sizes, baseline differences and measurement reliability to gauge the potential practical relevance of the observed changes.

## 3. Results

Baseline tensiomyography parameters by percussion condition are shown in Table 1. Td and Ts differed between duration conditions at baseline, whereas Tc, Tr and Dm did not differ significantly between frequency or duration levels (all *p* > 0.17), indicating broadly comparable baseline stiffness and displacement-related measures for the primary outcomes. The presence of baseline differences for selected secondary parameters underscores the importance of focusing on within-condition changes over time and on the pre-specified primary outcomes when interpreting duration-related effects.

Results of the linear mixed-effect models with time modeled as a continuous covariate are summarized in Table 2. These models showed a significant main effect of duration for Td (F (1,202.3) = 7.79, *p* = 0.006, η^2^p = 0.037) and Ts (F (1,309.9) = 13.07, *p* < 0.001, η^2^p = 0.040) and a small main effect of time for Tc (F(1,493.7) = 8.19, *p* = 0.004, η^2^p = 0.016), Ts (F(1,441.4) = 17.28, *p* < 0.001, η^2^p = 0.038), Tr (F(1,459.5) = 11.21, *p* < 0.001, η^2^p = 0.024) and Dm (F(1,1063.5) = 19.04, *p* < 0.001, η^2^p = 0.018), indicating systematic but small linear changes over the observation period in these parameters. Frequency showed no significant main effect on any TMG parameter (all *p* ≥ 0.14, η^2^p ≤ 0.013), and most higher-order interactions involving frequency or duration were non-significant. A duration × time interaction was observed for Tr (F (1,459.5) = 4.00, *p* = 0.046, η^2^p = 0.009) and Dm (F (1,1063.5) = 7.14, *p* = 0.008, η^2^p = 0.007), suggesting that the temporal evolution of relaxation time and maximal displacement slightly differed between the 3 and 6 min durations, although all associated effect sizes were small. Overall, these continuous-time models point to statistically detectable, small changes over time in all parameters and small duration-related effects on several secondary TMG measures, with only limited evidence for frequency-dependent trends and effect sizes that mostly fall within the range typically interpreted as small.

Estimated marginal means for percussion frequency and duration are presented in Table 3. For the primary outcomes, Dm was slightly higher on average in the 6 min than in the 3 min protocols (mean difference = −0.55 mm, 95% CI −0.97 to −0.14, *p* = 0.010, d = −0.39), whereas Tc showed no clear duration effect (mean difference = −0.38, 95% CI −1.08 to 0.32, *p* =0.285, d = −0.14). For the secondary parameters, Td (mean difference = −0.59 ms, 95% CI −1.05 to −0.13, *p* = 0.012, d = −0.34), Ts (mean difference = 12.48 ms, 95% CI 8.48 to 16.48, *p* < 0.001, d = 0.59), and Tr (mean difference = −10.48 ms, 95% CI −15.84 to −5.12, *p* < 0.001, d = −0.31) also differed between the 6 min and 3 min durations. Differences between 35 and 45 Hz were small and non-significant for all TMG parameters (all *p* ≥ 0.071, d ≤ 0.24), supporting the absence of main frequency effects observed in the mixed-model tests. It is important to note that, although Dm was higher on average in the 6 min compared with the 3 min protocols, Dm remained lower than baseline values throughout most of the post-intervention window, indicating that the duration effect reflects relative differences between two post-intervention conditions superimposed on an overall reduction from pre-intervention levels.

**Figure 1:**
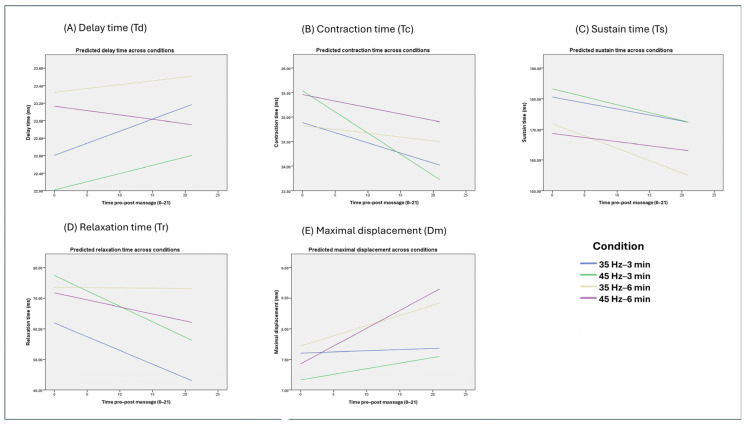
Predicted time-course of TMG parameters across percussion conditions. Predicted delay time (Td, (**A**)), contraction time (Tc, (**B**)), sustain time (Ts, (**C**)), relaxation time (Tr, (**D**)) and maximal displacement (Dm, (**E**)) across the four percussion protocols (35 Hz–3 min, 45 Hz–3 min, 35 Hz–6 min, 45 Hz–6 min). Time point 0 = baseline, time point 1 = immediately post-intervention, time points 2–21 = every 30 s thereafter.

Linear mixed-effect models with categorical time (Time_cat) are summarized in Table 4. Significant overall time effects were observed for all TMG parameters (Td, Tc, Ts, Tr and Dm (all *p* ≤ 0.015, η^2^p = 0.020–0.078)), indicating non-linear changes across discrete measurement points. For Td and Tr, neither the frequency × time_cat nor the frequency × duration × time_cat interactions reached significance (all *p* ≥ 0.105, η^2^p ≤ 0.013), indicating broadly similar time courses across frequency and duration conditions. Tc (F(21,2097.03) = 3.40, *p* < 0.001, η^2^p = 0.033) and Dm (F(21,2426.65) = 1.87, *p* = 0.010, η^2^p = 0.016) showed small frequency × time_cat interactions and Dm additionally exhibited small duration × time_cat and frequency × duration × time_cat interactions (all *p* ≤ 0.040, η^2^p = 0.014–0.015), indicating that the temporal evolution of maximal displacement, and to a lesser extent Tc, differed modestly between specific frequency–duration protocols. The time-course of Td, Tc, Ts, Tr and Dm across the four percussion conditions is illustrated in Figure 1.

Pairwise comparisons between baseline and post-intervention time points are presented in Table A1. For Td, significant increases relative to baseline were observed at time points 1–4 (all *p* < 0.001, d = 0.31–0.40), after which Td differences progressively diminished and were no longer significant following multiplicity correction. Tc was consistently higher than baseline across almost all post-intervention measurements (1–16 time points, most *p* < 0.001, d = 0.33–0.44), indicating a sustained but small elevation in contraction time. Ts showed a delayed pattern, with mainly non-significant changes early on, followed by widespread significant increases approximately from time point 8 onwards (*p* ≤ 0.05, d = 0.44–0.56), consistent with the categorical-time effect. For Tr, baseline differences were generally small and only sporadically reached significance (only at time point 18, *p* = 0.04, d = 0.47), whereas Dm was systematically reduced compared with baseline from time points 1 to 20 (all *p* < 0.001, d = −0.19 to −0.44), with the effect only disappearing at the final measurement. Overall, these findings indicate small but systematic short-term alterations in VM contractile behavior following PMT, with modest duration-related patterns that appear more consistent than frequency-related patterns within the constraints of the fixed design. Given the modest effect sizes (η^2^p mostly 0.02–0.08, d approximately 0.3–0.6) and the known measurement error of certain TMG parameters, these changes should be interpreted as subtle adjustments in contractile dynamics rather than large shifts in muscle function.

## 4. Discussion

To our knowledge, this is the first study to investigate the short-term effects of percussive massage therapy using different frequency–duration combinations on the VM contractile properties in non-fatigued muscle with tensiomyography and with such dense sampling over the first 10 min post-intervention. Across all conditions, PMT produced small but systematic changes in TMG-derived parameters, with transient increases in Td, a more sustained elevation in Tc, a delayed elevation in Ts and a persistent reduction in Dm, whereas Tr showed only sporadic changes over time. Within the constraints of the fixed design, duration showed more consistent patterns of association with several TMG responses than frequency. For example, 6 min protocols were associated with slightly higher Td, Ts, Tr and Dm than 3 min protocols when averaged across time, whereas differences between 35 and 45 Hz were statistically non-significant for estimated marginal means and frequency-related interactions were consistently small in magnitude. On the whole, these short-term dynamics provide a detailed, contractile-level description of how commonly used PMT settings modulate neuromuscular behavior in non-fatigued VM, while emphasizing that the absolute magnitudes of the observed changes are modest and should be interpreted in the context of measurement reliability, the fixed, non-randomized design and the absence of concurrent functional outcomes.

Devices such as Hypervolt and Hydragun massage guns applied for 1–5 min at approximately 40–60 Hz over the calf or hamstrings have consistently produced acute increases in joint range of motion and reductions in passive stiffness, typically without meaningful changes in maximal strength, a pattern that has been interpreted as reflecting an acute loosening effect rather than performance enhancement [9,15,36]. For example, Konrad et al. [9] and Ates et al. [36] both reported substantial dorsiflexion or hamstring flexibility gains after 5 min of percussive massage at around 53 Hz, with no decrement in maximal voluntary torque and suggested that these findings likely reflect decreased muscle stiffness and altered stretch tolerance rather than increased force-generating capacity. In line with this interpretation, our 35–45 Hz, 3–6 min VM protocols produced a pattern of reduced Dm together with increases in Tc and Ts, which is consistent with a slower and more prolonged contractile response, rather than with a TMG profile typically associated with explosive force production [28,34]. Although we did not measure joint range of motion or passive stiffness directly and cannot make strong inferences about flexibility in this sample, the observed TMG pattern resembles contractile signatures that have previously been associated with reduced stiffness and enhanced extensibility in other muscles and contexts [9,15,28,34,36]. These short-term TMG changes may also overlap with patterns observed in the early recovery phase after fatiguing exercise, where increased Tc and reduced Dm have been interpreted as indicators of altered muscle stiffness and contractile speed; however, any direct equivalence between PMT-induced and fatigue-induced responses remains speculative without concurrent fatigue and performance measures in the present study.

Other work has extended this general picture to different muscles and slightly longer treatments [15,16,21]. Bartik and Pacholek [21] reported that an 8 min Theragun protocol improved hamstring flexibility but did not enhance reactive or explosive strength indices, indicating that increased extensibility does not necessarily translate into acute improvements in stretch-shortening performance. Nevin et al. [16] similarly found that 5 min of percussive massage produced acute gains in passive knee extension comparable to static stretching, without clear superiority in performance outcomes, reinforcing the view that percussive massage behaves primarily as a flexibility-oriented intervention rather than a robust performance-enhancing intervention. Nakamura et al. [15] further showed that 120 s of gastrocnemius percussive massage at 53 Hz, with or without added heat, increased dorsiflexion range of motion and reduced passive stiffness without altering maximal voluntary isometric torque, again supporting a predominant effect on mechanical properties and stretch tolerance rather than maximal strength. Consistent with these findings, our data suggest that manipulating duration within a realistic clinical range (3 vs. 6 min) is associated with modest modulation of VM contractile behavior (particularly Dm and Tc, with additional changes in Ts and Tr), without any frequency-dependent facilitation of contractile speed or displacement that would suggest an acute performance enhancement [9,15,34,36]. Instead, the pattern of reduced Dm combined with slower and more prolonged contractions is congruent with an intervention that emphasizes extensibility and altered contraction dynamics over explosive capacity [28,37]. From a recovery perspective, such changes could be advantageous when the primary goal is to alleviate stiffness or perceived tightness, whereas they may be less desirable immediately before tasks requiring maximal rate of force development. However, these applied inferences remain tentative without direct performance data in the current study and should be interpreted as hypothesis-generating rather than definitive.

From an applied perspective, the present findings suggest that bouts of percussive massage to the VM at 35–45 Hz and 3–6 min are more likely to make the muscle exhibit a TMG-derived profile consistent with increased looseness (lower Dm and longer Tc and Ts) than to prime it for maximal explosiveness. In practical terms, such protocols may be best suited as part of recovery-oriented or lower-intensity technical sessions, where transient changes towards a slower and more prolonged contractile response are unlikely to compromise task demands and may align with goals of comfort and perceived reduction in stiffness [9,24,25]. Conversely, when very fast force production is critical, practitioners might consider the possibility that PMT-induced contractile changes persist for at least 10 min in non-fatigued VM and could interact with existing fatigue-related alterations in TMG parameters and therefore may wish to adjust the timing or dosage of PMT relative to key performance tasks. These applied considerations should be viewed as exploratory and require confirmation in studies that directly link PMT-induced TMG changes to task-specific performance outcomes.

Several limitations should be acknowledged when interpreting these findings. First, we examined only a single quadriceps muscle in young, physically active adults, so it remains unclear whether the observed frequency–duration effects generalize to other muscles, age groups or clinical populations. Secondly, by design, we focused exclusively on TMG-derived contractile parameters and did not include direct measures of joint range of motion, strength or explosive performance. This choice allowed precise characterization of neuromuscular responses but means that functional consequences of the observed pattern must be inferred from the prior literature rather than demonstrated within the present study and any causal interpretation linking TMG changes to flexibility or performance should therefore be considered tentative. Third, although our observation windows captured the first 10 min post-intervention, it is unknown whether different frequency–duration combinations might produce divergent responses over longer time frames under conditions of prior fatigue, where TMG kinetics may differ substantially. Fourth, all participants received the 35 Hz protocols in the first session and the 45 Hz protocols in the second session, with the right leg always assessed before the left and the 3 and 6 min durations assigned in a fixed manner within each session; randomization of protocol order and explicit modeling of leg dominance were therefore not implemented. As a result, subtle session-order or dominance-related influences on the observed responses cannot be fully excluded, and the fixed design and associated confounding of frequency with session and duration with leg side should be taken into account when extrapolating the present frequency–duration comparisons to other settings. Finally, several TMG parameters were analyzed, and multiple comparisons were performed across time points; although conservative corrections were applied, the risk of type I error inflation cannot be completely ruled out. Accordingly, the overall pattern and consistency of effects across parameters and time points, particularly for the primary outcomes Tc and Dm, should be given more weight than any single isolated comparison.

Future studies should integrate TMG with concurrent assessments of flexibility, force-velocity characteristics and task-specific performance, systematically randomize the order of frequency–duration combinations and their assignment to dominant and non-dominant legs, and extend these comparisons across muscles, age groups and clinical populations, including fatigued conditions. Such studies would allow a more direct linkage between PMT-induced changes in contractile properties, the time course of neuromuscular fatigue and recovery and meaningful functional outcomes and would help clarify which aspects of the present TMG patterns are robust across contexts and which are setting-specific.

## 5. Conclusions

In summary, clinically realistic PMT protocols applied to non-fatigued VM produced small but consistent alterations in TMG-derived contractile properties over the first 10 min post-intervention. These changes were characterized by transient increases in Td, a sustained elevation in Tc, delayed increases in Ts and a persistent reduction in Dm relative to baseline, whereas Tr showed only sporadic deviations. Within the constraints of the fixed, non-randomized design, application duration appeared more closely related to these alterations than frequency; however, duration effects were partially confounded with leg side and frequency with session order and should therefore be viewed as design-dependent associations rather than strong causal contrasts. Taken together, the data indicate that massage gun use at 35–45 Hz and 3–6 min is more likely to induce subtle, duration-sensitive modifications in VM contractile dynamics than to generate TMG patterns typically associated with acute enhancement of explosive performance. These findings should be considered exploratory, and future studies that combine TMG with functional measures of strength, rate of force development and range of motion, and that employ fully randomized designs, are needed to determine the practical significance of these contractile-level responses for recovery and performance.

## Figures and Tables

**Table 1:** Baseline tensiomyography parameters by percussion condition (pre-intervention).

Parameters	Condition	N	M ± SD	*p* (Frequency)	*p* (Duration)
Td	35 Hz–3 min	32	22.54 ± 0.34	0.681	<0.001
	45 Hz–3 min	32	22.37 ± 0.34		
	35 Hz–6 min	32	23.48 ± 0.39		
	45 Hz–6 min	32	23.45 ± 0.31		
Tc	35 Hz–3 min	32	24.96 ± 0.52	0.135	0.769
	45 Hz–3 min	32	26.21 ± 1.48		
	35 Hz–6 min	32	24.97 ± 0.58		
	45 Hz–6 min	32	25.79 ± 0.67		
Ts	35 Hz–3 min	32	182.28 ± 5.57	0.603	0.014
	45 Hz–3 min	32	185.63 ± 7.75		
	35 Hz–6 min	32	177.39 ± 5.28		
	45 Hz–6 min	32	169.73 ± 5.09		
Tr	35 Hz–3 min	32	63.81 ± 7.58	0.169	0.260
	45 Hz–3 min	32	87.47 ± 11.87		
	35 Hz–6 min	32	75.27 ± 7.97		
	45 Hz–6 min	32	72.66 ± 7.10		
Dm	35 Hz–3 min	32	7.57 ± 0.28	0.051	0.572
	45 Hz–3 min	32	7.13 ± 0.34		
	35 Hz–6 min	32	7.69 ± 0.25		
	45 Hz–6 min	32	7.27 ± 0.33		

M—mean, SD—standard deviation, *p* (frequency)—*p* values from linear mixed models testing baseline differences between frequencies (35 vs. 45 Hz), *p* (duration)—*p* values from linear mixed models testing baseline differences between durations (3 vs. 6 min).

**Table 2:** Linear mixed-model results with continuous time: type III tests of fixed effects for tensiomyography parameters.

Parameters	Factor	F	df_Effect	df_Error	*p*	η^2^p
Td	Frequency	0.851	1	202.263	0.357	0.004
	Duration	7.789	1	202.263	0.006	0.037
	Time	1.541	1	680.498	0.215	0.002
	Frequency × Duration	0.155	1	202.263	0.695	0.001
	Frequency × Time	0.577	1	680.498	0.448	0.001
	Duration × Time	1.709	1	680.498	0.192	0.003
	Frequency × Duration × Time	0.075	1	680.498	0.785	0.000
Tc	Frequency	1.841	1	168.061	0.177	0.011
	Duration	0.022	1	168.061	0.883	0.000
	Time	8.193	1	493.658	0.004	0.016
	Frequency × Duration	0.000	1	168.061	0.985	0.000
	Frequency × Time	0.878	1	493.658	0.349	0.002
	Duration × Time	2.084	1	493.658	0.150	0.004
	Frequency × Duration × Time	0.329	1	493.658	0.566	0.001
Ts	Frequency	0.007	1	309.897	0.933	0.000
	Duration	13.073	1	309.897	0.000	0.040
	Time	17.277	1	441.392	0.000	0.038
	Frequency × Duration	0.828	1	309.897	0.363	0.003
	Frequency × Time	0.745	1	441.392	0.388	0.002
	Duration × Time	0.110	1	441.392	0.740	0.000
	Frequency × Duration × Time	1.933	1	441.392	0.165	0.004
Tr	Frequency	2.181	1	373.820	0.141	0.006
	Duration	0.421	1	373.820	0.517	0.001
	Time	11.210	1	459.494	0.001	0.024
	Frequency × Duration	3.529	1	373.820	0.061	0.009
	Frequency × Time	0.594	1	459.494	0.441	0.001
	Duration × Time	3.995	1	459.494	0.046	0.009
	Frequency × Duration × Time	0.210	1	459.494	0.647	0.000
Dm	Frequency	2.116	1	166.654	0.148	0.013
	Duration	0.565	1	166.654	0.453	0.003
	Time	19.041	1	1063.516	0.000	0.018
	Frequency × Duration	0.088	1	166.654	0.767	0.001
	Frequency × Time	2.311	1	1063.516	0.129	0.002
	Duration × Time	7.136	1	1063.516	0.008	0.007
	Frequency × Duration × Time	0.153	1	1063.516	0.696	0.000

F—F statistics, df_effect—numerator degrees of freedom, df_error—denominator degrees of freedom, *p*—significance level, η^2^p—partial eta-squared.

**Table 3:** Estimated marginal means for percussion frequency and duration from linear mixed models.

Parameter	Factor	Level 1 (Mean ± SE)	Level 2 (Mean ± SE)	Mean Diff. (L1–L2)	95% CI Difference	*p*	d
Td	Frequency	35 Hz: 23.16 ± 0.29	45 Hz: 22.73 ± 0.29	0.42	−0.04, 0.88	0.071	0.242
	Duration	3 min: 22.65 ± 0.29	6 min: 23.24 ± 0.29	−0.59	−1.05, −0.13	0.012	−0.339
Tc	Frequency	35 Hz: 24.56 ± 0.474	45 Hz: 24.91 ± 0.47	−0.35	−1.05, 0.35	0.328	−0.127
	Duration	3 min: 24.54 ± 0.47	6 min: 24.92 ± 0.47	−0.38	−1.08, 0.32	0.285	−0.138
Ts	Frequency	35 Hz: 169.92 ± 4.28	45 Hz: 171.81 ± 4.28	−1.89	−5.89, 2.11	0.353	−0.090
	Duration	3 min: 177.11 ± 4.28	6 min: 164.63 ± 4.28	12.48	8.48, 16.48	0.000	0.593
Tr	Frequency	35 Hz: 62.94 ± 5.24	45 Hz: 66.89 ± 5.24	−3.95	−9.31, 1.41	0.148	−0.118
	Duration	3 min: 59.67 ± 5.24	6 min: 70.15 ± 5.24	−10.48	−15.84, −5.12	0.000	−0.313
Dm	Frequency	35 Hz: 7.86 ± 0.26	45 Hz: 7.70 ± 0.262	0.16	−0.26, 0.58	0.456	0.112
	Duration	3 min: 7.50 ± 0.26	6 min: 8.05 ± 0.26	−0.55	−0.97, −0.14	0.010	−0.385

SE—standard error, CI—confidence interval for mean difference (Level 1–Level 2), *p*—Bonferroni-adjusted *p* values, d—Cohen’s d, frequency levels—35 and 45 Hz, duration levels—3 and 6 min.

**Table 4:** Linear mixed-model results with categorical time: overall time and interaction effects (type III tests of fixed effects).

Parameter	Effect	F	df_Effect	df_Error	*p*	η^2^p
Td	Time_cat	9.192	21	2279.23	0.000	0.078
	Frequency × Time_cat	1.347	21	2279.23	0.133	0.012
	Duration × Time_cat	2.108	21	2279.23	0.002	0.019
	Frequency × Duration × Time_cat	1.403	21	2279.23	0.105	0.013
Tc	Time_cat	6.852	21	2097.03	0.000	0.064
	Frequency × Time_cat	3.401	21	2097.03	0.000	0.033
	Duration × Time_cat	0.564	21	2097.03	0.943	0.006
	Frequency × Duration × Time_cat	1.173	21	2097.03	0.265	0.012
Ts	Time_cat	1.791	21	1817.549	0.015	0.020
	Frequency × Time_cat	0.586	21	1817.549	0.930	0.007
	Duration × Time_cat	0.893	21	1817.549	0.602	0.010
	Frequency × Duration × Time_cat	1.023	21	1817.549	0.431	0.012
Tr	Time_cat	2.046	21	1658.377	0.003	0.025
	Frequency × Time_cat	0.961	21	1658.377	0.510	0.012
	Duration × Time_cat	0.728	21	1658.377	0.808	0.009
	Frequency × Duration × Time_cat	1.028	21	1658.377	0.425	0.013
Dm	Time_cat	4.261	21	2426.647	0.000	0.036
	Frequency × Time_cat	1.869	21	2426.647	0.010	0.016
	Duration × Time_cat	1.603	21	2426.647	0.040	0.014
	Frequency × Duration × Time_cat	1.782	21	2426.647	0.016	0.015

Time_cat—categorical time factor (baseline and 21 post-intervention time points), F—F statistics, df_effect—numerator degrees of freedom, df_error—denominator degrees of freedom, *p*—significance level, η^2^p—partial eta-squared.

## Data Availability

The data presented in this study are available on request from the corresponding author.

## Abbreviations

The following abbreviations are used in this manuscript:

PMT	Percussive massage therapy
TMG	Tensiomyography
VM	Vastus medialis
Dm	Maximal displacement
Tc	Contraction time
Td	Delay time
Ts	Sustain time
Tr	Relaxation time
LMM	Linear mixed-effect model
EMM	Estimated marginal mean

## Appendix A

**Table A1:** Pairwise comparisons between baseline and post-intervention time points for tensiomyography parameters.

Parameter	Times Comparison	Mean Diff.	SE	95% CI Lower	95% CI Upper	*p*	d
Td	0 vs. 1	0.700	0.055	0.496	0.904	<0.001	0.403
	0 vs. 2	0.590	0.077	0.303	0.871	<0.001	0.339
	0 vs. 3	0.570	0.092	0.232	0.916	<0.001	0.328
	0 vs. 4	0.540	0.105	0.147	0.924	<0.001	0.311
	0 vs. 5	0.396	0.115	−0.320	0.923	0.14	0.228
	0 vs. 6	0.287	0.124	−0.174	0.748	1.00	0.165
	0 vs. 7	0.244	0.132	−0.246	0.734	1.00	0.140
	0 vs. 8	0.159	0.139	−0.357	0.675	1.00	0.091
	0 vs. 9	0.095	0.145	−0.440	0.635	1.00	0.055
	0 vs. 10	0.140	0.151	−0.419	0.700	1.00	0.081
	0 vs. 11	0.088	0.156	−0.490	0.667	1.00	0.051
	0 vs. 12	0.053	0.161	−0.543	0.649	1.00	0.030
	0 vs. 13	0.047	0.165	−0.564	0.658	1.00	0.027
	0 vs. 14	0.050	0.169	−0.575	0.676	1.00	0.029
	0 vs. 15	−0.100	0.172	−0.648	0.628	1.00	−0.058
	0 vs. 16	0.024	0.175	−0.627	0.674	1.00	0.014
	0 vs. 17	−0.130	0.178	−0.791	0.632	1.00	−0.075
	0 vs. 18	−0.094	0.181	−0.766	0.577	1.00	−0.054
	0 vs. 19	−0.166	0.183	−0.847	0.515	1.00	−0.095
	0 vs. 20	−0.121	0.186	−0.811	0.659	1.00	−0.070
	0 vs. 21	−0.178	0.188	−0.876	0.619	1.00	−0.102
Tc	0 vs. 1	1.114	0.095	0.763	1.465	<0.001	0.406
	0 vs. 2	1.188	0.132	0.701	1.676	<0.001	0.433
	0 vs. 3	1.134	0.158	0.549	1.720	<0.001	0.413
	0 vs. 4	1.151	0.179	0.488	1.814	<0.001	0.419
	0 vs. 5	1.216	0.196	0.489	1.944	<0.001	0.443
	0 vs. 6	1.141	0.211	0.359	1.924	<0.001	0.415
	0 vs. 7	1.127	0.224	0.297	1.957	<0.001	0.410
	0 vs. 8	1.099	0.235	0.227	1.970	<0.001	0.400
	0 vs. 9	1.069	0.245	0.160	1.977	<0.001	0.389
	0 vs. 10	1.000	0.254	0.059	1.940	0.02	0.364
	0 vs. 11	0.995	0.262	0.025	1.965	0.03	0.362
	0 vs. 12	0.940	0.269	−0.057	1.936	0.11	0.342
	0 vs. 13	0.942	0.275	−0.078	1.963	0.15	0.343
	0 vs. 14	0.916	0.281	−0.126	1.958	0.26	0.333
	0 vs. 15	0.933	0.286	−0.129	1.994	0.26	0.340
	0 vs. 16	0.978	0.29	−0.101	2.057	0.18	0.356
	0 vs. 17	0.967	0.295	−0.129	2.062	0.25	0.352
	0 vs. 18	0.948	0.299	−0.162	2.059	0.36	0.345
	0 vs. 19	0.987	0.302	−0.136	2.111	0.26	0.359
	0 vs. 20	0.945	0.305	−0.191	2.082	0.47	0.344
	0 vs. 21	1.033	0.308	−0.115	2.181	0.20	0.376
Ts	0 vs. 1	2.755	1.183	−1.628	7.139	1.00	0.131
	0 vs. 2	5.089	1.587	−0.788	10.966	0.31	0.242
	0 vs. 3	5.729	1.846	−1.110	12.568	0.45	0.272
	0 vs. 4	5.077	2.03	−2.442	12.597	1.00	0.241
	0 vs. 5	6.406	2.165	−1.617	14.428	0.72	0.305
	0 vs. 6	7.965	2.267	−0.439	16.368	0.11	0.379
	0 vs. 7	8.475	2.345	−0.221	17.172	0.07	0.403
	0 vs. 8	9.166	2.406	0.241	18.090	0.03	0.436
	0 vs. 9	10.377	2.453	1.274	19.480	0.01	0.493
	0 vs. 10	9.278	2.49	0.035	18.522	0.05	0.441
	0 vs. 11	11.414	2.52	2.058	20.769	<0.001	0.543
	0 vs. 12	11.029	2.543	1.586	20.473	<0.001	0.524
	0 vs. 13	10.964	2.561	1.450	20.478	0.01	0.521
	0 vs. 14	11.265	2.575	1.695	20.835	<0.001	0.536
	0 vs. 15	10.093	2.587	0.478	19.708	0.02	0.480
	0 vs. 16	9.555	2.596	−0.096	19.205	0.06	0.454
	0 vs. 17	10.964	2.603	1.285	20.643	0.01	0.521
	0 vs. 18	11.761	2.609	2.059	21.463	<0.001	0.559
	0 vs. 19	10.608	2.614	0.888	20.329	0.13	0.504
	0 vs. 20	11.380	2.617	1.645	21.115	<0.001	0.541
	0 vs. 21	11.431	2.62	1.684	21.178	<0.001	0.543
Tr	0 vs. 1	7.827	2.299	−0.689	16.343	0.16	0.234
	0 vs. 2	9.562	2.999	−1.546	20.670	0.33	0.285
	0 vs. 3	6.156	3.405	−6.458	18.771	1.00	0.184
	0 vs. 4	5.666	3.664	−7.909	19.241	1.00	0.169
	0 vs. 5	6.607	3.835	−7.606	20.820	1.00	0.197
	0 vs. 6	7.863	3.951	−6.784	22.510	1.00	0.235
	0 vs. 7	8.600	4.03	−6.345	23.545	1.00	0.257
	0 vs. 8	10.755	4.085	−4.397	25.907	1.00	0.321
	0 vs. 9	12.918	4.122	−2.379	28.214	0.41	0.385
	0 vs. 10	7.110	4.149	−8.288	22.509	1.00	0.212
	0 vs. 11	12.558	4.167	−2.912	28.027	0.61	0.375
	0 vs. 12	10.529	4.18	−4.991	26.049	1.00	0.314
	0 vs. 13	13.917	4.189	−1.638	29.472	0.21	0.415
	0 vs. 14	9.671	4.196	−5.909	25.251	1.00	0.289
	0 vs. 15	11.974	4.2	−3.624	27.571	1.00	0.357
	0 vs. 16	13.442	4.203	−2.168	29.052	0.33	0.401
	0 vs. 17	14.299	4.205	−1.319	29.918	0.16	0.427
	0 vs. 18	15.807	4.207	0.182	31.431	0.04	0.472
	0 vs. 19	14.711	4.208	−0.919	30.430	0.11	0.439
	0 vs. 20	14.717	4.209	−0.916	30.349	0.11	0.439
	0 vs. 21	14.690	4.209	−0.944	30.324	0.12	0.438
Dm	0 vs. 1	−0.275	0.036	−0.406	−0.143	<0.001	−0.192
	0 vs. 2	−0.362	0.05	−0.547	−0.178	<0.001	−0.253
	0 vs. 3	−0.447	0.06	−0.671	−0.224	<0.001	−0.313
	0 vs. 4	−0.462	0.067	−0.718	−0.207	<0.001	−0.323
	0 vs. 5	−0.444	0.076	−0.727	−0.162	<0.001	−0.311
	0 vs. 6	−0.484	0.083	−0.791	−0.178	<0.001	−0.339
	0 vs. 7	−0.453	0.089	−0.781	−0.125	<0.001	−0.317
	0 vs. 8	−0.475	0.094	−0.822	−0.127	<0.001	−0.332
	0 vs. 9	−0.483	0.099	−0.848	−0.118	<0.001	−0.338
	0 vs. 10	−0.540	0.103	−0.921	−0.159	<0.001	−0.378
	0 vs. 11	−0.515	0.107	−0.911	−0.119	<0.001	−0.360
	0 vs. 12	−0.567	0.111	−0.974	−0.157	<0.001	−0.397
	0 vs. 13	−0.576	0.114	−0.999	−0.154	<0.001	−0.403
	0 vs. 14	−0.581	0.117	−1.016	−0.147	<0.001	−0.407
	0 vs. 15	−0.585	0.12	−1.03	−0.139	<0.001	−0.409
	0 vs. 16	−0.551	0.123	−1.007	−0.095	<0.001	−0.386
	0 vs. 17	−0.584	0.126	−1.05	−0.118	<0.001	−0.409
	0 vs. 18	−0.631	0.128	−1.106	−0.156	<0.001	−0.442
	0 vs. 19	−0.599	0.13	−1.083	−0.115	<0.001	−0.419
	0 vs. 20	−0.613	0.133	−1.106	−0.121	<0.001	−0.429
	0 vs. 21	−0.607	0.135	−1.107	−0.107	0.20	−0.425

Mean diff.—estimated mean difference between baseline (time 0) and the indicated time point, SE—standard error, 95% CI—95% confidence interval for mean difference, *p*—Bonferroni-adjusted *p* value, d—Cohen’s d.
